# Decision - delivery interval and perinatal outcome of emergency caesarean sections at a tertiary institution 

**DOI:** 10.12669/pjms.305.5470

**Published:** 2014

**Authors:** Onyedikachi Edwin Chukwudi, Chukwunwendu Anthony Okonkwo

**Affiliations:** 1 Dr. Onyedikachi Edwin Chukwudi, MBBS, Department of Obstetrics & Gynaecology, University of Benin Teaching Hospital, Benin City, Nigeria.; 2 Dr. Chukwunwendu Anthony Okonkwo, MBBS, FMCOG, FICS, D.MAS, Department of Obstetrics & Gynaecology, University of Benin Teaching Hospital, Benin City, Nigeria.

**Keywords:** Decision to Delivery interval, Caesarean Section, Perinatal outcome

## Abstract

***Background and objective:*** A decision-to-delivery interval (DDI) of 30 minutes for emergency Caesarean sections (CS) has been widely recommended, but there is little evidence to support it. Recent studies however, have questioned not only the practicability of this target but also its anticipated beneficial effect on neonatal outcome and medico-legal implications. Our objective in this study was to find out the time between decision-delivery interval and perinatal outcome of emergency caesarean section at a tertiary care institution in Nigeria

***Methods:*** This was a retrospective study of cases of emergency Caesarean section performed over a 12-month period. Relevant data were collected from the labour ward and theatre records and case files of the University of Benin Teaching Hospital, Benin City, Nigeria between January 1 and December 31, 2012.

***Results:*** A total of 352 emergency Caesarean sections done during the period were reviewed. Only 20 (5.7%) of these were performed within the recommended 30 minutes DDI. The mean DDI was 106.3 + 79.5 minutes and there was no significant correlation between DDI and perinatal outcome. The major causes of delay were anaesthetic delay and busy theatre suits.

***Conclusion:*** This study demonstrated a lack of correlation between DDI and perinatal outcome, which may indicate decision delivery interval of 30 minutes or less may not be applicable to all emergency CS, especially in developing countries with infrastructural challenges. However when faced with acute or catastrophic foetal or maternal conditions, expedited delivery is indicated.

## INTRODUCTION

In present day Obstetric practice, the need to avoid the adverse neonatal effects of perinatal asphyxia has been one of the common indications for Caesarean section. Expeditious delivery is dependent on decision to perform Caesarean delivery and time lines achieved.^1^ Since the dawn of Caesarean births, operative deliveries have been performed in extreme clinical situations.^2^^-^^4^

In modern Obstetrics, routine Caesarean deliveries are offered electively to women for variety of indications; or performed in emergency foetal or maternal complications or both.^5^ Caesarean section has been classified based on the severity of foetal and/or maternal situation into emergency, urgent, scheduled and elective Caesarean deliveries.^6^ According to the classification, emergency CS is performed in situations that are extremely life-threatening for the mother or foetus or both. Some authors refer to this category as ‘crash’ Caesarean delivery.^1^ Urgent Caesarean delivery is the one performed for maternal or fetal compromise which is not immediately life-threatening. Scheduled CS is done in situations needing early delivery but no maternal or fetal compromise while elective CS is done at a time to suit the mother and the maternity team.^6^

The decision-to-delivery interval (DDI) is defined as the interval in minutes from the date and time of decision to carry out Caesarean section to the date and time of delivery of the baby.^1^^,^^7^ It is not synonymous with decision-to-incision time where the goal of the birth of a baby is yet to be achieved.^1^ A decision-to-delivery interval of 30 minutes for emergency Caesarean section has been widely recommended,^8^^,^^9^ but there is little evidence to support it.^10^ Inability to meet this target has been the basis for medico-legal suits.^11^ The ‘30 minute rule’ for a DDI takes its origin from the Guidelines to perinatal care jointly developed by the American Academy of Paediatrics and the American College of Obstetricians and Gynaecologists.^12^ Recent studies however, have cast doubts not only on the practicability of this target but also on its anticipated beneficial effect on neonatal outcome.^4^^,^^13^^-^^16^

Our objective in this study was to find out the time between decision-delivery interval and perinatal outcome of emergency caesarean section at a tertiary care institution in Nigeria.

## METHODS

This was a retrospective study of cases of emergency Caesarean section between January 1 and December 31, 2012 at the University of Benin Teaching Hospital, Benin City, Nigeria. It included cases of emergency C/S done for booked parturients with live singleton gestation between 37 and 42 weeks. Unbooked parturients and those with hypertensive disorders were excluded to minimize bias due to pre-existing foetal compromise and delays from stabilization and the need for more detailed investigations. Relevant data were collected from the labour ward and theatre records and patients’ case files.

Data collected included sociodemograhic characteristics such as age, parity, and gestational age at delivery as well as the indication for CS, date and time of decision for CS and date and time of delivery. The DDI was derived as the interval between decision and delivery of the baby.

Perinatal parameters such as sex, birth weight, Apgar scores and stillbirth (if any) as well as the need for admission into special care baby unit (SCBU) were also obtained. Other parameters obtained included the reason for delay, timing of surgery, rank of the surgeon, type of anesthesia used and maternal complications encountered, if any.

Data entry and analysis was done using the Statistical package for social sciences, SPSS (IBM SPSS statistic 20). Group mean was compared using ANOVA and the distribution of data was examined using the likelihood chi square test. A probability level of ≤ 0.05 was considered statistically significant.

## RESULTS

A total of 352 emergency Caesarean sections were performed for indications other than hypertensive disorders, on parturients at between 37 and 42 weeks of gestation, whose pregnancies were booked. Of the 352 emergency CS cases, ten (2.8%) were done by consultants, 63.4% (223) by senior registrars and 119 (33.8%) by registrars. Forty-eight percent of the surgeries were done during the day while 52.0% were at night. Majority of the cases (97.2%) were done under spinal anaesthesia while 2.8% was done under general anaesthesia. None of the cases was done under epidural anaesthesia. No stillbirth or maternal death was recorded in this study, however two babies suffered early neonatal death.

Seventy percent of the parturients were between 25 – 34 years, teenage pregnancy accounted for 1.1 percent of the study population. More than half (58.2%) of the parturients were nulliparous (para 0) while 15 (4.3%) were grandmultiparous.

The commonest indication was cephalopelvic disproportion in labour (41.5%) followed by fetal distress (27.8%). Others were cervical dystocia (6.0%), 2 or more previous CS in labour (4.3%), nullipara breech in labour (4.3%), ante partum haemorrhage (placenta praevia - 3.7%, abruptio placentae – 3.4%), footling breech in labour (2.8%) and cord prolapse (1.4%).

The overall mean decision to delivery interval was 106.3 79.5 minutes. Cord prolapse had the shortest mean DDI (23.4 2.3 minutes) followed by fetal distress (68.7 39.7 minutes) and abruptio placentae with live baby (76.5 48.7 minutes). Bleeding placenta praevia had the longest mean DDI (198.7 88.0 minutes). These differences in mean DDI were statistically significant (p=0.000).

Two hundred and fifty-eight babies (73.3%) had 1^st^ minute Apgar score of 7 or greater, only 2.0% (7) had a score of 3 or less. The babies with 1^st^ minute Apgar scores of <3 had the shortest mean DDI (72.1 42.5minutes) while those with 1^st^ minute Apgar scores of >7 had longest mean DDI. This is not statistically significant (p= 0.091).

Fifty-three babies (15.0%) were admitted into the SCBU for birth asphyxia while 85.0% were not. The mean DDI for the admitted babies (69.8 33.1 minutes) was significantly shorter than that of those not admitted (112.8 83.6 minutes, p= 0.000). Two of the admitted babiess died a few days later.

Out of the 352 cases of emergency CS reviewed, 20 (5.7%) were performed within 30 minutes DDI. Thirty four percent (34.1%) of the cases were done within 60 minutes and about 35% remained undone after 100 minutes. Anaesthetic delay and busy theatre suits contributed over 80% of the delays. Other reasons for delay included lack of blood for transfusion (8.8%) and delay in transfer to theatre (4.3%). Lack of blood for transfusion was responsible for the longest delays (mean DDI= 183.9 94.5 minutes) while delay in transfer to theatre resulted in the shortest delays (mean DDI= 61.6 22.7 minutes). This was statistically significant (p= 0.000).

## DISCUSSION

The mean decision to delivery interval from this study (106 minutes) is still a far cry from the recommended 30 minutes. It confirms the findings from other studies^4^^,^^14^, which have shown that the current recommendation of 30 minutes interval between decision and emergency Caesarean delivery is difficult to achieve in practice. However, the result from this study is an improvement from results obtained in similar studies from other Nigerian centres,^15^^,^^16^ reported mean DDI of 200 minutes and 252 minutes respectively. Preterm and post term pregnancies with their characteristic increased risk of perinatal morbidity and mortality were also excluded. These were done to minimize bias, and may have contributed to the much shorter DDI obtained as well as low incidence of perinatal morbidity and mortality recorded in this study.

**Table-I:** Socio-demographic characteristic of the Study Population

*Variable*	*Number*	*Percentage (%)*
Age(Years)		
**15 – 19** **20 – 24** **25 – 29** **30 – 34** **35 – 39** ** 40**	**4** **50** **147** **114** **31** **6**	**1.1** **14.2** **41.8** **32.4** **8.8** **1.7**
Total	352	100.0
		
Parity		
**0** **1-4** **5**	**205** **132** **15**	**58.2** **37.5** **4.3**
Total	352	100.0

**Table-II:** Indications for emergency caesarean section

*Indication*	*Number*	*Percentage (%)*
**Foetal distress** **Cephalopelvic disproportion in labour** * **APH (Placenta praevia)** * **APH (Abruptio placentae)** **2 or more previous CS in labour** **Cervical dystocia** **Cord prolapse** **Footling breech in labour** **Nullipara breech in labour** ** **Others**	**98** **146** **13** **12** **15** **21** **5** **10** **15** **17**	**27.8** **41.5** **3.7** **3.4** **4.3** **6.0** **1.4** **2.8** **4.3** **4.8**
Total	352	100.0

* *APH- ante partum haemorrhage. *

** *Others=1 previous CS+poor progress & abnormal lie in labour.*

**Table-III:** Mean DDI according to indications

*Indications*	*N (%)*	*Mean DDI SD (min)*
**Fetal distress**	**98 (27.8)**	**68.7 39.7**
**Cephalopelvic disproportion in labour**	**146 (41.5)**	**120.0 84.9**
* **APH (Placenta praevia)**	**13 (3.7)**	**198.7 88.0**
* **APH (Abruptio placentae)**	**12 (3.4)**	**76.5 48.7**
**2 or more previous CS in labour**	**15 (4.3)**	**178.8 108.1**
**Cervical dystocia**	**21 (6.0)**	**97.4 56.6**
**Cord prolapsed**	**5 (1.4)**	**23.4 2.3**
**Footling breech in labour**	**10 (2.8)**	**123.8 122.1**
**Nullipara breech in labour **	**15 (4.3)**	**125.8 79.6**
**Others	**17 (4.8) **	**99.4 30.0**
Total	353 (100.0)	106.3 79.5

* *APH = antepartum haemorrhage. Others=1 previous CS+poor progress & abnormal lie in labour. P=0.000*

**Table-IV:** One-minute Apgar scores Versus DDI

*1-minute Apgar score*	*N (%)*	*Mean DDI SD (minutes)*
**<** **3** **4-5** **6** **>** **7**	**7 (2.0)** **61 (17.3)** **26 (7.4)** **258 (73.3)**	**72.1 42.5** **95.1 56.0** **101.6 93.2** **110.4 81.2**
Total	352 (100.0)	106.3 79.5

*N=number, %= percent, SD= standard deviation. P= 0.091*

**Table-V:** SCBU Admission versus Mean DDI

*SCBU Admission*	*N (%)*	*Mean DDI (minutes)*	*SD*
**Yes**	**53 (15.0)**	**69.8**	**33.1**
**No**	**299 (85.0)**	**112.8**	**83.6**
Total	352 (100.0)	106.3	79.5

*SCBU= special care baby unit. SD= standard deviation, N= number, %= percent. P= 0.000*

**Table-VI:** Reasons for delay versus DDI

*Reasons for delay*	*N (%)*	*Mean DDI SD*
* **No delay**	**20 (5.7)**	**25.0 3.2**
**Anaesthetic delay**	**173 (49.1)**	**72.7 39.5**
**Delay in transfer to theatre**	**15 (4.3)**	**61.6 22.7**
**Busy theatre suits**	**110 (31.3)**	**157.3 88.0**
**Lack of blood for transfusion**	**31 (8.8)**	**183.9 94.5**
** **Others**	**3 (0.9)**	**139.0 1.3**
Total	352 (100.0)	106.3 79.5

* *DDI within 30 minutes,*

** *Initial objection to surgery and hesitation to give consent. P= 0.000.*

Kolas et al.^10^ and Sayegh et al.^17^ in separate studies in Europe reported mean DDI of 39.5 minutes and 52.4 minutes respectively. This huge difference between the DDI obtained in the Nigerian studies and those from their foreign counterparts may be a reflection of improved facilities and more effective co-ordination of services in those parts of the world. Only 5.7% of the Caesarean deliveries in the present study were performed within 30 minutes of DDI. This along with the findings of the other Nigerian studies^15^^,^^16^, in which none of the CS was performed within 30 minutes of decision, suggest that the recommended 30 minutes DDI for emergency CS is currently not feasible in Nigeria.

There was no correlation between the 1^st^ minute Apgar score of delivered babies and mean DDI as well as between mean DDI and Neonatal intensive care admission. Although, this is contrary to findings from some studies^7^^,^^18^, several other studies^13^^-^^15^ similarly could not demonstrate any correlation between mean DDI and perinatal outcome. In the present study, the mean DDI of the fifty-three babies admitted into the SCBU out of which two later died, was 69 minutes, and that of the seven babies with 1^st^ minute Apgar scores of 3 or less, all of whom survived, was 72 minutes. This clearly suggests that perinatal morbidities and mortality recorded may be unrelated to DDI.

It has been suggested that a DDI of 30 minutes or less may not be applicable to all emergency CS but when faced with an acute or catastrophic fetal or less commonly maternal conditions, expedited delivery is warranted and any purposeful delay is unjustifiable.^1^ In this study such conditions included cord prolapse, fetal distress and abruptio placentae. These indications contributed the shortest mean DDI. Similar findings were reported by other studies.^10^^,^^13^ Seniority of the surgeon was not a significant predictor in this study. This is similar to the finding of Mackenzie and Cooke^13^, though contrary to that of Kolas et al.^10^ in Norway. Caesarean sections performed at night were significantly quicker than those performed during the day. This may not be unconnected to busy theatre suits that are characteristic of working hours due to the inclusion of elective cases. This was supported by the Norwegian study.

The major causes of delay in the present study were anaesthetic delay, which has been universally published,^1^^,^^10^ and busy theatre suits. Over 97.2% of cases reviewed were done using spinal anaesthesia. This is because it has been found to be safer and the technique of choice.^18^ There was no evidence to suggest that adverse perinatal outcome resulted from delays occasioned by spinal anaesthesia as these occurred only in a few cases of multiple needle attempts. Most anaesthetic delays were a result of too few anaesthetists available, being mostly engaged elsewhere in the hospital when required for emergency CS and having to be waited for. Only 2.8% of the Caesarean sections were done under general anaesthesia, usually for potential bleeding cases (APH), acute fetal conditions or when spinal anaesthesia failed.

Despite lack of correlation between DDI and perinatal outcome, unnecessary long DDI is not justified just as litigation on the ground of DDI is not justified. A decision delivery interval of 30 minutes or less may not be applicable to all emergency CS, but when faced with acute or catastrophic fetal or maternal conditions, expedited delivery is warranted and any purposeful delay is unjustified.

## Author‘s Contribution:

***Dr O.E.Chukwudi: ***Conceived, designed and did statistical analysis

***Dr C.A.Okonkwo: ***Did review and final approval of manuscript, takes responsibility for all aspects of the work.

## References

[1] Rashid N, Nalliah S (2007). Understanding the Decision-Delivery Interval in Cesarean Births. IeJSME.

[2] Cunningham G, Gant NF, Leveno KJ, Gilstrap LC, Hauth JC, Wenstrom KD (2001). Williams Obstetrics.

[3] Lagrew DC, Bush MC, McKeown AM, Lagrew NG (2006). Emergent (crash) cesarean delivery: Indications & outcomes. Am J Obstet Gynecol.

[4] Spencer MK, MacLennan AH (2001). How long does it take to deliver a baby by emergency caesarean section?. Australian & New Zealand J Obstet Gynaecol.

[5] (2004). ACOG: Surgery & Patient choice, ethics of decision making: ACOG committee opinion: Nov. 2003;289. Int J Gynaecol Obstet.

[6] Lucas DN, Yentis SM, Kinsella SM, Holdcroft A, May AE, Wee M (2000). Urgency of caesarean section: a New classification. J Royal Soc Med.

[7] Thomas J, Paranjothy S, James D (2004). National cross sectional survey to determine whether the decision to delivery interval is critical in emergency caesarean section. BMJ.

[8] Hannah WJ, Basket TM, Chance GW, Hamilton EF, Huchcroft S, King FK (1986). Indications for caesarean section: Final statement of the Panel of the National consensus conference on aspects of caesarean birth. Can Med Assoc J.

[9] Maternal & Child Health Research Consortium (2000). Confidential Enquiry into Stillbirths & Deaths in infancy. 7th Annual Report.

[10] Kolas T, Hofos D, Olan P (2006). Predictions for the decision-to-delivery interval for emergency Cesarean section in Norway. Acta Obstetricia et Gynecologica.

[11] Anonymous (2003). Delay in performing caesarean section. http://www.tmlt.org/customer/cases/case%209.html.

[12] Guidelines for Perinatal care (2002).

[13] Mackenzie IZ, Cooke I (2002). What is a reasonable time from decision-to-delivery by caesarean section? Evidence from 415 deliveries. BJOG.

[14] Helmy WH, Jolaoso AS, Ifaturoti OO, Afify SA, Jones MH ( 2002). The decision-to-delivery interval for emergency caesarean section: Is 30 min a realistic target?. Int J Obstet Gynaecol.

[15] Onah HE, Ibeziako N, Umezulike AC, Effetie ER, Ogbuokiri CM (2005). Decision-delivery interval & Perinatal outcome in emergency caesarean section. J Obstet Gynaecol.

[16] Fyneface-Ogan S, Mato CN, Enyindah CE (2009). Decision to delivery Interval: Reasons for delay. J Med Biomedical Res.

[17] Sayegh I, Dupuis O, Clement HJ, Rudigoz RC (2004). Evaluating the decision to delivery in emergency caesarean sections. Euro J Obstets Gynaecol Reprod Biol.

[18] Singh R, Deo S, Pradeep Y (2012). The decision-to-delivery interval in emergency Caesarean sections and its correlation with perinatal outcome: evidence from 204 deliveries in a developing country. Trop Doct.

